# ALK2 Receptor Kinase Association with FKBP12.6 Is Structurally Conserved with the ALK2-FKBP12 Complex

**DOI:** 10.3390/biomedicines9020129

**Published:** 2021-01-29

**Authors:** Eleanor Williams, Elise Riesebos, Georgina Kerr, Alex N. Bullock

**Affiliations:** 1 Centre for Medicines Discovery, University of Oxford, Old Road Campus Research Building, Roosevelt Drive, Oxford OX3 7DQ, UK; eleanor.williams@cmd.ox.ac.uk (E.W.); elise_riesebos@hotmail.com (E.R.); georgina.kerr@medsci.ox.ac.uk (G.K.); 2 Department of Clinical Genetics, Amsterdam UMC, Vrije Universiteit Amsterdam, 1081 BT Amsterdam, The Netherlands

**Keywords:** BMP, kinase, FKBP12, FKBP12.6, ACVR1, ALK2, fibrodysplasia ossificans progressiva

## Abstract

The immunophilin FKBP12 is a known inhibitor of type I BMP and TGF-β receptors that competes for binding with their substrate SMADs. FKBP12 and the close paralog FKBP12.6 additionally assemble with ryanodine receptors to control Ca^2+^ release. Binding of FKBP12.6 to BMP/TGF-β receptors has yet to be investigated, but appears plausible given its high sequence similarity to FKBP12. Here, we found that FKBP12.6 can assemble with BMP and TGF-β-family type I receptors, but not with type II receptors. Cellular immunoprecipitation confirmed similar binding of FKBP12 and FKBP12.6 to the BMP receptor ALK2 (ACVR1), a known target of mutations in the congenital syndrome fibrodysplasia ossificans progressiva (FOP), as well as the pediatric brain tumor diffuse intrinsic pontine glioma (DIPG). SEC-MALS analyses using purified proteins indicated a direct 1:1 interaction between FKBP12.6 and the receptor’s cytoplasmic domains. The 2.17 Å structure of this ALK2-FKBP12.6 complex bound to the inhibitor dorsomorphin showed FKBP12.6 binding to the GS domain of ALK2 in a manner equivalent to the FKBP12 complex, with ALK2 residues Phe198 and Leu199 extending into the FK506-binding pocket of FKBP12.6. These findings suggest a level of redundancy in FKBP-family regulation of BMP and TGF-β signaling.

## 1. Introduction

Activin receptor-like kinases (ALK1-7) are a group of type I receptor serine/threonine kinases that phosphorylate SMAD transcription factors in response to the binding of secreted ligands of the transforming growth factor-beta (TGF-β) family, such as TGF-β, nodal, activins, growth differentiation factors (GDFs) and the bone morphogenetic proteins (BMPs) [1,2]. These signal transduction pathways are critical for embryogenesis, immunity, and tissue repair and present active areas of drug development for cancer and musculoskeletal diseases [3,4,5]. Much recent interest has focused on the BMP receptor kinase ALK2, encoded by the gene *ACVR1*, due to the discovery that gain of function mutations in the receptor’s intracellular domain associate with the rare congenital syndrome fibrodysplasia ossificans progressiva (FOP), as well as the pediatric brain tumor diffuse intrinsic pontine glioma (DIPG) [6,7]. These mutations confer neomorphic activity, allowing ALK2 to signal in response to activins, as well as BMPs [8,9].

The cytosolic immunophilin FKBP12 was identified by its binding to the immunosuppressant drugs FK506 (Tacrolimus), and rapamycin (Sirolimus) [10]. It is now recognized as only one of 15 human FK506-binding proteins (FKBPs) [10]. In the absence of TGF-β family ligands, FKBP12 can also bind to the intracellular domains of ALK1-7 to prevent leaky signaling [11]. Crystal structures of FKBP12 bound to ALK2, ALK5 (TGFRB1) and ALK6 (BMPR1B) have been solved and revealed its binding to two helices in the glycine-serine rich (GS) domain of ALKs that occurs N-terminal to the kinase domain [12,13,14]. This assembly forces the GS loop into an inaccessible conformation that prohibits the GS site phosphorylations essential for kinase activation and the recruitment of the substrate SMADs [12,14,15]. The inhibitory activity of FKBP12 towards ALKs is therefore dependent on its protein-protein interactions and independent of its prolyl isomerase activity. Secreted TGF-β family ligands induce a heterotetrameric complex of type I receptors and constitutively active type II receptors. This assembly triggers the release of FKBP12 and the transphosphorylation of the ALK1-7 GS loop by the type II receptor, allowing signal transduction to commence [15,16,17].

Dissociation of FKBP12 from the type I receptor GS domain can also be induced using small molecules such as FK506 that compete for binding to the same hydrophobic pocket in FBKP12. A chemical screen identified FK506 as a molecule capable of enhancing BMP signaling in pulmonary artery endothelial cells expressing a dysfunctional mutant BMPR2 [18]. However, siRNA-mediated depletion of FKBP12 was insufficient to phenocopy the activation of BMP receptors in these cells [18]. Similarly, siRNA treatments to individually reduce the levels of FKBP12.6, FKBP13, FKBP25, FKBP52, FKBP54, and FKBP38 were also insufficient to induce BMP signaling, consistent with a redundancy in FKBPs as binding partners of the type I receptors [18].

Loss or destabilization of the FKBP12 interaction is also observed for the ALK2 mutants found in FOP and DIPG [14,19,20,21], and has been suggested to contribute to their acquired activin-responsiveness [8]. Indeed, several reports have shown that wild-type ALK2 can acquire similar activin responsiveness upon FK506 treatment [8,22].

The binding of BMP/TGF-β receptors to FKBPs is therefore of interest for the understanding of disease mechanisms, as well as for the development of drug intervention strategies. Here we confirmed the binding of FKBP12.6 to type I receptors using purified recombinant proteins. We also solved the structure of ALK2 in complex with FKBP12.6, revealing its high conservation with the equivalent FKBP12 complex. These data support a level of redundancy in FKBP-family binding to ALK2 in agreement with the observed effects of published siRNA screening.

## 2. Experimental Section

### 2.1. Plasmids

All constructs were prepared by ligation-independent cloning. Full-length human FKBP12 (FKBP1A) and FKBP12.6 (FKBP1B) were cloned into pNIC28-Bsa4 for bacterial expression [23]. The intracellular domains of human ALK2 (a.a. 172–499), ALK5 (a.a. 162–503), ALK6 (a.a. 168–502), and ActRIIA (a.a. 191–488) were cloned into pFB-LIC-Bse for baculoviral expression [23]. For immunoprecipitation, full-length human ALK2 was cloned into pcDNA3 with a C-terminal FLAG tag as previously described [14]. Full length human FKBP12 and FKBP12.6 were cloned into pcDNA3 with an N-terminal GFP tag.

### 2.2. Immunoprecipitation

MDA-MB-231 BRE-Luc/TK Renilla cells (ximbio #156439) were a kind gift from Caroline Hill and form an established reporter cell line for BMP signaling [24]. The cell line was originally derived by transfecting the human breast cancer cell line MDA-MB-31 with plasmids encoding BMP responsive element (BRE)-Luciferase, a Renilla reporter driven by the thymidine kinase (TK) promoter, and pRetroSuper for puromycin resistance. Cells were plated using 6 × 10^5^ cells per 10 cm dish. Cells were transfected the following day with FLAG-tagged ALK2 and GFP-tagged FKBP12 or FKBP12.6 by FuGENE (Promega, Madison, WI, USA), following the manufacturer’s protocol. After a further 24 h, the cells were lysed for 1 h at 4 °C in lysis buffer containing 20 mm Tris-HCl, pH 7.5, 150 mm NaCl, 25 mM NaF, 0.1% Triton X-100, and protease inhibitors (Roche Applied Science, Penzberg, Germany). Lysates were clarified by centrifugation, and the protein concentrations were measured using the BCA assay (Pierce, Appleton, WI, USA). 1 mg of lysate was incubated for 2 h at 4 °C with anti-FLAG M2 beads (Sigma #A2220, St. Louis, MO, USA) before the beads were washed thoroughly in lysis buffer and resuspended in loading dye for SDS PAGE and immunoblotting. PVDF membrane was probed with relevant antibodies; these antibodies were rabbit anti-GFP antibody (Sigma Aldrich #G1544) and HRP-conjugated anti-rabbit (Sigma #A0545) or anti-FLAG-HRP (Sigma #A8592). Bands were detected by ECL (Pierce) and images acquired on a LAS-4000 image analyzer.

### 2.3. SEC-MALS

Analytical size exclusion chromatography combined with multi-angle light scattering (SEC-MALS) was performed using a Shodex KW-803 column on a Dionex micro-HPLC system that was coupled to the analytics of a Tetra detector (Malvern Instruments Ltd., Malvern, UK). Proteins at 5 mg/mL were buffered in 50 mM HEPES, pH 7.5, 300 mM NaCl, 0.5 mM TCEP and applied to the column at a flow rate of 0.5 mL/min.

### 2.4. Protein Expression and Purification

FKBP12 and FKBP12.6 proteins were recombinantly expressed in *Escherichia coli* strain BL21(DE3)R3-pRARE2 [25]. Cultures in LB media were grown at 37 °C to OD_600_ = 0.5 before the overnight induction of protein expression at 21 °C by the addition of 0.4 mM isopropyl 1-thio-β-d-galactopyranoside (IPTG). The intracellular domains of BMP/TGF-β receptor family kinases were expressed in Sf9 insect cells [26]. Baculoviral expression was performed at 27 °C with shaking at 110 rpm and cells harvested at 48 h post infection. Harvested cells were resuspended in binding buffer (50 mM HEPES, pH 7.5; 500 mM NaCl; 5% glycerol; 5 mM imidazole) supplemented with protease inhibitor cocktail set V (Calbiochem) and 0.5 mM tris(2-carboxyethyl)phosphine (TCEP). Cells were then lysed by ultrasonication and treated with 0.15% polyethylenimine (PEI) to precipitate nucleic acids. Supernatants were recovered after further centrifugation at 4 °C for 1 h. Proteins were applied by gravity flow onto nickel-sepharose columns, washed with binding buffer containing 30 mM imidazole and eluted by a step gradient of similar buffers containing up to 250 mM imidazole. The N-terminal hexahistidine tags were then cleaved at 4 °C using tobacco etch virus (TEV) protease and purified further by size exclusion chromatography using a S200 HiLoad 16/60 Superdex column run on an ÄKTA-Express system buffered in 50 mM HEPES, pH 7.5, 300 mM NaCl, 0.5 mM TCEP. For crystallization of the ALK2-FKBP12.6 complex, the two proteins were initially purified separately by nickel affinity chromatography and then mixed for further purification by size exclusion chromatography with FKBP12.6 in slight excess.

### 2.5. Crystallization

Crystals of the ALK2-FKBP12.6 complex were grown at 4 °C using the sitting-drop vapor diffusion method. The purified protein complex was buffered in 50 mM HEPES, pH 7.5, 150 mM NaCl, and 10 mM DTT. Kinase inhibitor dorsomorphin (Calbiochem) was added to a final concentration of 1 mM and the protein complex concentrated to 7.5 mg/mL (calculated using an extinction co-efficient of 61,880 M^−1^cm^−1^). Diffracting crystals formed in 150 nL drops, mixing 100 nL of protein solution with 50 nL of a reservoir solution containing 1.8 M ammonium citrate pH 7.2. On mounting, crystals were cryoprotected with mother liquor plus 25% ethylene glycol and vitrified in liquid nitrogen.

### 2.6. Structure Determination

Diffraction data were collected at the Diamond Light Source, station I04 using monochromatic radiation at wavelength 0.9686 Å. Data were processed with MOSFLM [27] and scaled using the program AIMLESS [28]. Initial phases were obtained by molecular replacement using the program PHASER [29] and the structure of the ALK2-FKBP12 complex (PDB ID 3H9R [14]) as a search model. Structure refinement was performed using REFMAC5 [30] from the CCP4 suite and manually rebuilt with COOT [31]. Diffraction data collection and refinement statistics are shown in Table 1.

### 2.7. Accession Numbers

The atomic coordinates and structure factors have been deposited in the Protein Data Bank with PDB ID 4C02 (http://wwpdb.org/).

## 3. Results

### 3.1. FKBP12.6 Binds to Type I Receptors

**Figure 1:**
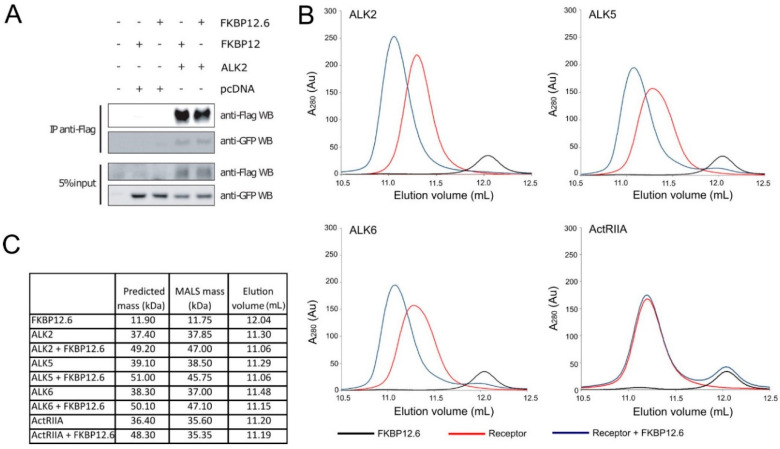
FKBP12.6 binds to type I receptors in a 1:1 complex. (**A**) Immunoprecipitation (IP) to assess ALK2 binding to FKBP12 or FKBP12.6 in MDA-MB-231 cells transfected with FLAG-tagged ALK2 and GFP-tagged FKBPs. Proteins were detected by Western blot (WB) using the indicated antibodies. (**B**) Analytical size exclusion chromatography elution profiles for the indicated individual recombinant proteins and upon mixing with FKBP12.6. (**C**) Multi-angle light scattering (MALS) data for the peak of the indicated elution samples.

FKBP12.6 is the closest paralog to FKBP12 sharing 83.3% sequence identity over 108 amino acids, whereas other family members share only 40–50% identity [10]. We hypothesized that FKBP12.6 was the most likely FKBP family protein to bind to ALK2 outside of the known interaction of FKBP12. To investigate this binding, we first performed an immunoprecipitation experiment in human MDA-MB-231 cells using FKBP12 as a known ligand for comparison. Cells were transiently co-transfected with FLAG-tagged ALK2 and GFP-tagged FKBP12.6 or FKBP12. After 24 h of expression, the binding of the FKBPs was assessed by FLAG immunoprecipitation of ALK2. As shown by Western blot in Figure 1A, the immunoprecipitation of ALK2 resulted in the recovery of both FKBP12 and FKBP12.6 at comparable levels. These data indicate that FKBP12.6 can bind to ALK2 in cells in a similar manner to FKBP12.

To confirm that FKBP12.6 bound to ALK2 through a direct interaction, we purified the two individual recombinant proteins and investigated their complex formation in vitro using analytical size exclusion chromatography combined with multi-angle light scattering (SEC-MALS). For these experiments, we used a construct expressing the ALK2 intracellular residues 172–499, which contain the receptor’s GS and kinase domains, as well as a construct for full length FKBP12.6. When mixed and analyzed by SEC-MALS, the two proteins eluted as a single peak eluting at a lower elution volume than either protein alone indicating a shift to higher molecular mass (Figure 1B). Their assembly into an ALK2-FKBP12.6 complex of 1:1 stoichiometry was further confirmed by a MALS measurement of 47.0 KDa, in excellent agreement with the expected mass of the complex of 49.2 KDa (Figure 1C).

We then used SEC-MALS to investigate whether FKBP12.6 could also bind to other type I or type II receptors. For these experiments, we purified recombinant proteins for the intracellular domains of the type I BMP receptor ALK6 (BMPR1B), the type I TGF-β receptor ALK5 (TGFBR1) and the type II receptor ActRIIA (ACVR2A). When mixed with FKBP12.6, both type I receptors exhibited a shift to lower elution volume in the SEC profiles, as previously observed for ALK2 (Figure 1B). The MALS measurements indicated that ALK5 and ALK6 were also eluting as a 1:1 complex with FKBP12.6, as judged by their apparent molecular mass shift (Figure 1C). By contrast, the type II receptor ActRIIA showed no change in elution volume, or MALS mass, when mixed with FKBP12.6, consistent with a lack of binding due to the absence of a GS domain in this receptor class. Together, these data reveal that FKBP12.6 can bind directly to type I receptors, but not to type II receptors, as previously found for FKBP12 [11,12].

### 3.2. Structure Determination of ALK2 Complexed with FKBP12.6

To understand the structural basis of the ALK2-FKBP12.6 interaction, we co-purified FKBP12.6 with the intracellular region of ALK2 containing the GS and kinase domains (residues 172–499) to perform crystallization experiments. The crystallization of protein kinases is often hindered by the flexible association of their N and C-lobes, which are tethered by a single hinge region. To overcome this difficulty, we also incubated the protein sample with the small molecule inhibitor dorsomorphin [32]. A previous structure of the ALK2-FKBP12 complex bound to dorsomorphin showed this inhibitor packing at the interface between the N and C-lobes where its interactions within the ATP-binding site helped to stabilize one specific conformation of the kinase domain [14]. Any structure of an ALK2-FKBP12.6-dorsomorphin complex would therefore be directly comparable with this previous work. Crystal screens were set up at 20 °C as well as 4 °C with four distinct crystallization coarse screens (HCS, HIN1, JCSG and LFS) [33]. Diffracting crystals were obtained at 4 °C using a precipitant containing 1.8 M ammonium citrate pH 7.2 and the resulting structure was solved at a resolution of 2.17 Å in space group *P* 4_1_ 3 2.

### 3.3. Overview of the Structure of the ALK2-FKBP12.6 Complex

**Figure 2:**
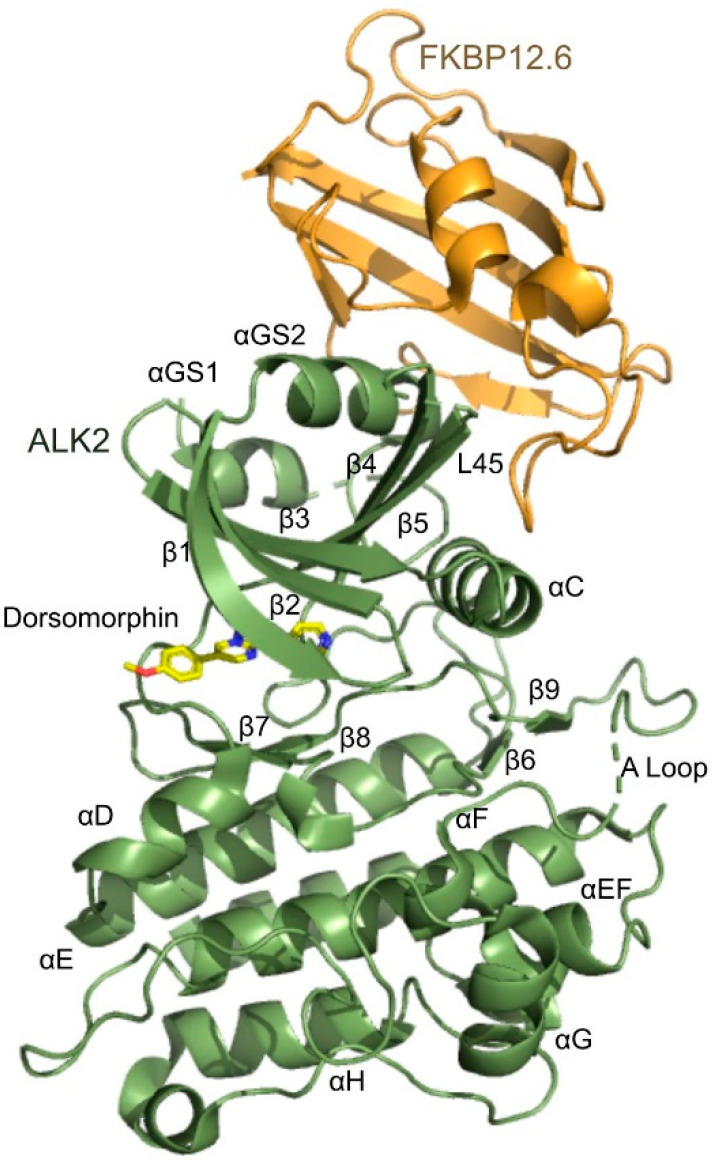
Structure of the ALK2-FKBP12.6 complex with dorsomorphin. ALK2 (green) and FKBP12.6 (orange) are shown in ribbon representation with secondary structural elements labelled. Regions missing in the electron density maps, where the chain could not be modeled, are shown by dashed lines. The kinase inhibitor dorsomorphin bound in the ATP-binding site is shown in a stick representation with its carbon, oxygen, and nitrogen atoms displayed in yellow, red, and blue, respectively.

The structure solution revealed a single ALK2-FKBP12.6 complex in the crystal’s asymmetric unit with both proteins exhibiting their known folds (Figure 2). The electron density maps allowed the FKBP12.6 chain to be modeled across the full length of the protein. The ALK2 chain was modeled from Ser178 to Asp499, except for apparent regions of disorder at the C-terminus of αGS1 (residues His186-Thr189), the tip of the β4-β5-hairpin (L45 loop, His274-Ser275) and part of the kinase activation loop (A-loop, Gly371-Asn373). ALK2 displays the expected bilobal kinase fold with two additional helices, αGS1 and αGS2, that form the N-terminal GS domain. The GS loop lies between the two αGS helices and must be phosphorylated by the type II receptor for ALK2 kinase activation. FKBP12.6 sits atop the GS domain of ALK2 with its binding centered on the αGS2 helix (Figure 2). As reported previously for the ALK2-FKBP12 structure [14], the binding of FKBP12.6 forces the GS loop into a buried position between the β4-strand and αC helix where it is inaccessible to the type II receptor. In this manner, FKBPs can protect against leaky ALK2 signaling until BMP ligands engage the receptor’s extracellular domains and the resulting complex with type II receptors causes FKBPs to be displaced. The burial of the GS loop ensures the kinase domain of ALK2 exhibits an inactive conformation in which the catalytic salt bridge between Lys235 (β3) and Glu248 (αC) is broken. The structure also shows dorsomorphin bound to the kinase ATP-binding site as previously observed with a single hydrogen bond to the hinge residue His286 [14].

**Figure 3:**
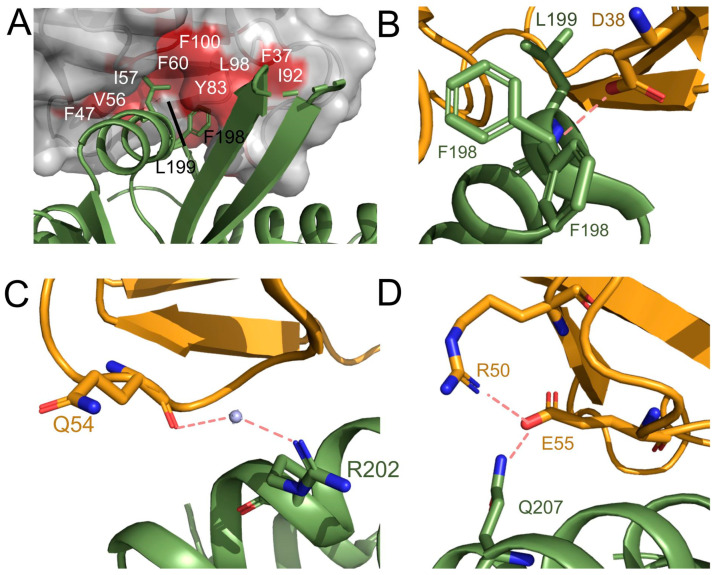
Interactions between ALK2 and FKBP12.6. (**A**) αGS2 residues ALK2 Phe198 and Leu199 (green) inserted into a hydrophobic pocket in FKBP12.6 (grey surface with hydrophobic patch shown in red). (**B**) Backbone hydrogen bond from ALK2 Leu199 to the side chain of FKBP12.6 Asp38. (**C**) Water-mediated hydrogen bond between ALK2 Arg202 and FKBP12.6 Gln54. (**D**) Hydrogen bonding between ALK2 Gln207 and FKBP12.6 Glu55 and Arg50.

The interface between ALK2 and FKBP12.6 shows the interaction of two hydrophobic residues on the surface of ALK2 (Phe198 and Leu199) extending into a hydrophobic pocket in FKBP12.6 that is alternatively used to bind to FK506 (Figure 3A). This interaction is further supported by a hydrogen bond between the backbone amide of Leu199 and the side chain of FKBP12.6 Asp38 (Figure 3B). The αGS2 helix of ALK2 forms additional electrostatic interactions with FKBP12.6 through a water-mediated hydrogen bond between the backbone carbonyl of ALK2 Arg202 and FKBP12.6 Gln54 (Figure 3C), as well as a direct hydrogen bond between the side chains of ALK2 Gln207 and FKBP12.6 Glu55 (Figure 3D). Of note, all four ALK2 residues (Phe198, Leu199, Arg202, and Gln207) are closely associated with sites of mutation found in individuals with FOP.

### 3.4. Comparison with the ALK2-FKBP12 Complex

**Figure 4:**
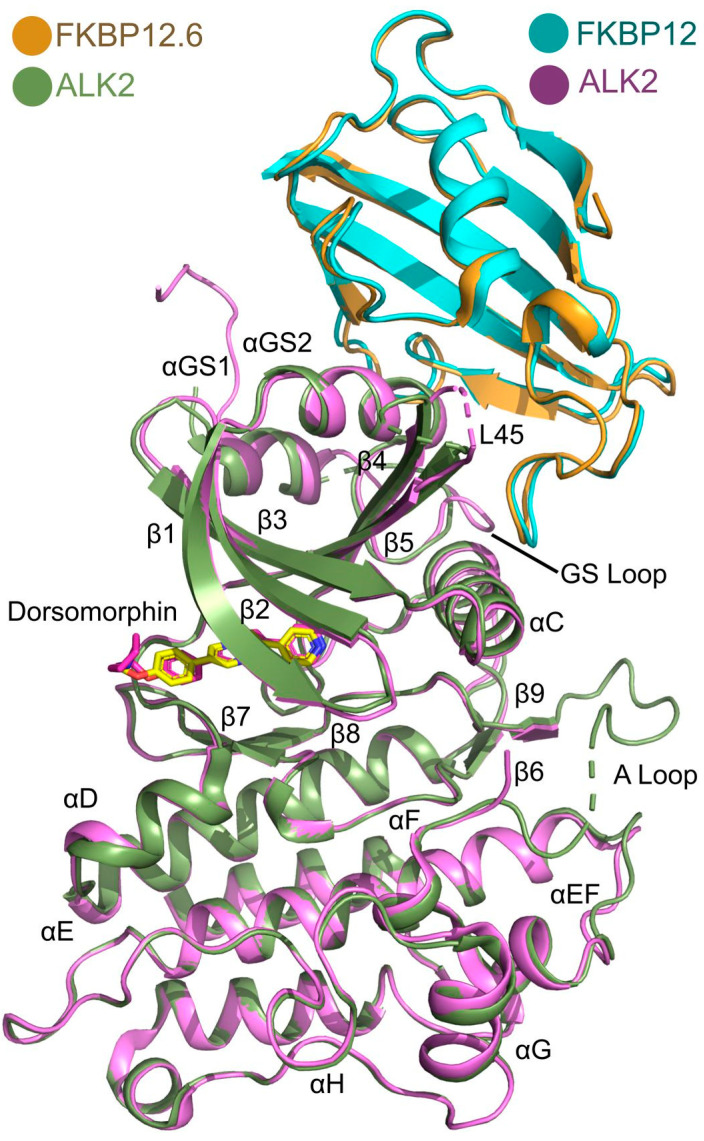
Structural comparison of ALK2 binding to FKBP12 and FKBP12.6. Superposition reveals high structural conservation between the ALK2-FKBP12.6-dorsomorphin complex (green/orange/yellow) and the previously reported structure of the ALK2-FKBP12-dorsomorphin complex (purple/cyan/magenta)(PDB 3H9R [14]).

The structural basis of ALK2 binding to FKBP12.6 can be compared to the previous structural analyses of the ALK2-FKBP12 complex (Figure 4). The latter complex crystallized with dorsomorphin in space group *P* 2_1_ 2_1_ 2_1_ using a precipitant containing 30% PEG3350, 0.25 M ammonium sulfate, 0.1 M BisTris pH 6 and the structure was solved at a resolution of 2.35 Å. Despite differences in space group (orthorhombic vs. cubic) and crystallization condition (high vs. low salt), superposition of the ALK2-FKBP12 and ALK2-FKBP12.6 complexes shows high structural similarity with a Cα rmsd of 0.68 Å over 408 residues. Structural conservation is particularly evident at the binding interface with both FKBPs packing similarly against the αGS2 helix (Figure 4). In fact, minor differences in the main chain were largely restricted to flexible regions outside the complex interface, such as the ALK2 kinase activation loop (A-loop, Figure 4).

**Figure 5:**
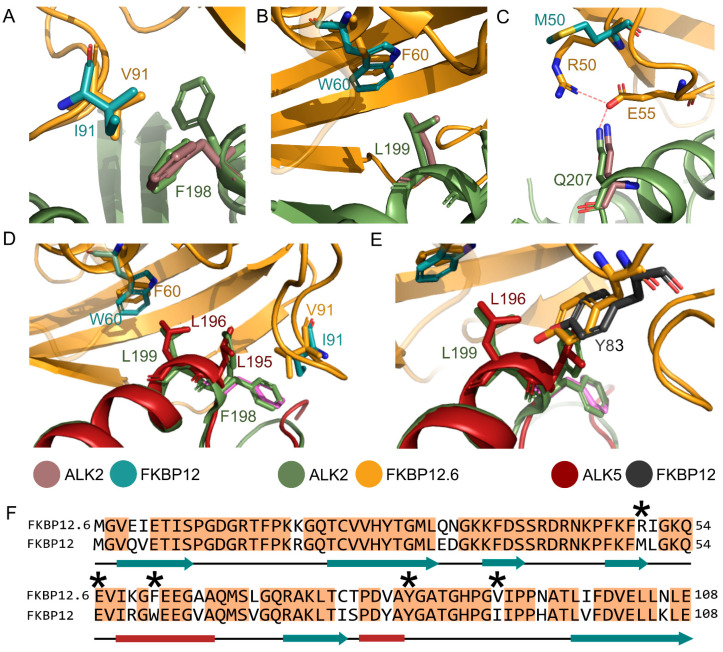
Structural variations between ALK2 binding to FKBP12 and FKBP12.6. Structures of ALK2-FKBP12 (PDB 3H9R [14]), ALK2-FKBP12.6 (PDB 4C02), and ALK5-FKBP12 (1B6C [12]) were superimposed and are displayed in the indicated colors. Different views of the FKBPs binding to the αGS2 helices of ALK2 and ALK5 are shown highlighting selected side chain and conformational differences. (**A**) FKBP12 Ile91 is replaced by Val91 in FKBP12.6, which packs against two distinct conformations of ALK2 Phe198. (**B**) FKBP12 Trp60 is replaced by Phe60 in FKBP12.6 and packs against ALK2 Leu199. (**C**) FKBP12.6 Glu55 and Arg50 can form an intramolecular interaction in addition to Glu55 interaction with ALK2 Gln207. (**D**) ALK2 Phe198-Leu199 are replaced by Leu195-Leu196 in ALK5. Both ALK5 leucines occupy the hydrophobic FK506-binding pocket in the FKBP12 co-structure (red). In the equivalent ALK2-FKBP12 structure, ALK2 Phe198 (magenta) folds outside this pocket. The ALK2-FKBP12.6 co-structure shows ALK2 Phe198 (green) adopting both conformations. (**E**) The conserved FKBP residue Tyr83 packs more closely to the αGS2 helix in the ALK2 co-structures compared to the ALK5 co-structure. (**F**) Sequence alignment of FKBP12 and FKBP12.6 highlighting conserved residues and secondary structural elements. Side chains displayed in the figure are denoted by an asterisk.

FKBP12.6 is distinguished from FKBP12 by several notable side chain substitutions at the binding interface with ALK2 (Figure 5). As referenced above, the most significant interactions from ALK2 are the hydrophobic contacts formed by Phe198 and Leu199. Their interaction partners in FKBP12, Ile91 and Trp60, are replaced by smaller hydrophobics in FKBP12.6 Val91 and Phe60, respectively (Figure 5A,B). The substitution of FKBP12 Met50 with FKBP12.6 Arg50 also confers an additional potential intramolecular interaction for FKBP12.6 Glu55, which may compete with its binding of ALK2 Gln207 (Figure 5C). While the larger hydrophobic residues in FKBP12 may be favorable, the impact on ALK2 binding is likely to be marginal.

Structural comparisons can also be drawn with the ALK5-FKBP12 complex. In ALK5, Leu195 and Leu196 replace ALK2 Phe198 and Leu199, respectively, and pack efficiently in the FK506-binding pocket of FKBP12 (Figure 5D,E). By contrast, Phe198 folds outside this pocket in the equivalent ALK2-FKBP12 complex [14], which might suggest that its bulkier side chain is excluded due to steric hindrance. However, Phe198 adopts both conformations in the new ALK2-FKBP12.6 co-structure to mimic ALK5 Leu195 inside the pocket, as well as the previously observed conformation outside the pocket (Figure 5A,D,E). Of note, both ALK2 co-structures show closer packing of the loop containing FKBP12/12.6 Tyr83 than the equivalent ALK5 complex (Figure 5E). Other hydrophobic interface residues in FKBP12.6 are strictly conserved with FKBP12 (Figure 3A).

Overall, the structure of the ALK2-FKBP12.6 complex fully supports the observation that FKBP12.6 can interact with type I receptors in a similar fashion to FKBP12.

## 4. Discussion

FKBP12 was the first FKBP-family protein to be identified and remains highly studied due to its pleiotropic functions in many important biological systems [10]. The binding of its hydrophobic pocket to the drugs FK506 and rapamycin acts to induce gain of function interactions with calcineurin and mammalian target of rapamycin (mTOR), respectively. Conversely, these drugs inhibit its direct protein-protein interactions with the ryanodine receptors (RyR1 and RyR2) and the activin receptor-like kinases (ALK1-7), which all compete for the same hydrophobic binding pocket [10]. The paralog FKBP12.6 appears to show overlapping, but non-redundant functions. Its interactions are best characterized in the context of the ryanodine receptors, which control Ca^2+^ release in muscle cells. While FKBP12 and FKBP12.6 are known components of the RyR1 and RyR2 complexes, FKBP12.6 appears to have a dominant role in controlling RyR2 activity in cardiac muscle [10].

Here, we demonstrated that FKBP12 and FKBP12.6 also share the capability to bind to the activin receptor-like kinases, both in cells and in vitro. Our FKBP12.6 binding studies using purified components in solution revealed a direct 1:1 interaction with BMP as well as TGF-β receptors that was confirmed by the 2.17 Å structure of the ALK2-FKBP12.6 complex. The structure shows 1:1 binding of FKBP12.6 to the GS domain of ALK2 in a manner essentially identical to that of FKBP12, consistent with their high sequence identity of 83.3%. The binding of FKBP12 and FKBP12.6 to the GS domain also explains their failure to bind to the type II receptors in TGF-β family signaling, which lack an equivalent GS domain region.

Closer analyses of the ALK2-FKBP12.6 structure revealed four residues in ALK2 mediating key hydrophobic or electrostatic interactions with FKBP12.6. These four residues, Phe198, Leu199, Arg202 and Gln207, are closely associated with sites of mutation in individuals with FOP (namely P197_F198delinsL, R202I, and Q207E) [14,34]. The latter mutation Q207E is also found in children with the brain tumor DIPG [6]. Each mutation destabilizes the inactive conformation of ALK2 bound by FKBPs and promotes kinase activation [14].

The GS domain plays a dual role in signal transduction since it mediates interactions with substrate SMADs, as well as inhibitory proteins such as FKBP12 or FKBP12.6 [15]. In the absence of ligand stimulation, the interactions of the intracellular GS domain take on an inhibitory function to protect against any unwanted leaky signaling. The ALK2 complexes with FKBP12 and FKBP12.6 show how these proteins can shield the GS domain from the constitutively active type II receptors and force the GS loop into a buried conformation that is inaccessible for transphosphorylation. Upon ligand stimulation, type II receptors are brought into a stable complex with the type I receptors, which favors GS loop phosphorylation over the binding of FKBPs. In turn, this phosphorylation promotes the binding of SMAD transcription factors which can then be phosphorylated by the type I receptor for translocation to the nucleus [13]. The structural mechanisms of SMAD-receptor binding are yet to be elucidated, but the shared ancestral fold of the SMAD/FHA domain superfamily suggests a similar phosphopeptide interaction [15].

A recent study showed that FKBP12.6 also forms a tight interaction with the protein glomulin, which was previously recognized as a partner for the FK1 domain of FKBP51 [35]. Based on the crystal structure of FKBP51 [36], we predict that this domain will also be compatible with the binding of ALK1-7, assuming some flexibility of the FKBP51 loop regions, as previously observed for the FKBP family [10]. Thus, in future it would be of interest to test this interaction, as well as other FKBPs. Many FKBPs are widely expressed, although six FKBP proteins localise to the endoplasmic reticulum, including FKBP13, FKBP19, FKBP22, FKBP23, FKBP60, and FKBP65 [37], which would separate them from the cytoplasmic GS domains of ALK1-7. To assess the potential redundancy between FKBPs for binding of ALKs, it will be important to develop new assays to measure the relative affinities of these interactions. For example, while many FKBPs can bind to FK506 and rapamycin, their affinities can vary considerably [10].

Overall, TGF-β family signaling is composed of multiple components at every level, including multiple ligands, receptors, receptor-activated SMADs (R-SMADs), and inhibitory SMADs (SMAD6/7) [2]. The commonalities between FKBP12 and FKBP12.6 suggest that multiple components are also found at the level of the FKBPs. We hope these insights will stimulate researchers to consider both FKBP12 and FKBP12.6 when investigating the gain of function mechanisms of ALK2 in FOP and DIPG, as well as other type I BMP/TGF-β receptors.

## Figures and Tables

**Table 1:** Diffraction data and refinement statistics.

PDB Accession Code	PDB ID: 4C02
**Data collection**	
Beamline	Diamond I04
Wavelength (Å)	0.9686
Resolution (Å)	39.79–2.17
Spacegroup	*P* 4_1_ 3 2
Cell dimensions	*a* = 182.33, *b* = 182.33, *c* = 182.33 Å
	α = 90°, β = 90°, γ = 90.0°
No. unique reflections	54946
Completeness ^1^ (%)	99.8 (99.7)
I/σI ^1^	8.0 (2.0)
R_merge_^1^	0.17 (1.07)
Redundancy ^1^	9.0 (9.3)
**Refinement**	
No. refinement atoms:	
Macromolecules	3271
Ligands	179
Waters	231
R_fact_ (%)	17.73
R_free_ (%)	19.75
rms deviation bond ^2^ (Å)	0.0069
rms deviation angle ^2^ (°)	1.5390
Ramachandran favour	97.4%
Ramachandran allowed	99.7%

^1^ Values in parenthesis refer to the highest resolution shell. ^2^ Rms indicates root-mean-square.

## Data Availability

The atomic coordinates and structure factors have been deposited in the Protein Data Bank with PDB ID 4C02 (http://wwpdb.org/).
